# Changes in Diversity and Abundance of Ammonia-Oxidizing Archaea and Bacteria along a Glacier Retreating Chronosequence in the Tianshan Mountains, China

**DOI:** 10.3390/microorganisms11122871

**Published:** 2023-11-27

**Authors:** Xiukun Wu, Wei Zhang, Guangxiu Liu, Tuo Chen, Zhongqin Li

**Affiliations:** 1 Key Laboratory of Desert and Desertification, Northwest Institute of Eco-Environment and Resources, Chinese Academy of Sciences, Lanzhou 730000, China; 2 Key Laboratory of Extreme Environmental Microbial Resources and Engineering, Lanzhou 730000, China; 3 State Key Laboratory of Cryospheric Sciences, Northwest Institute of Eco-Environment and Resources, Chinese Academy of Sciences, Lanzhou 730000, China

**Keywords:** AOA and AOB, community structure, glacier foreland, chronosequence, Tianshan No. 1 Glacier

## Abstract

Glaciers retreating due to global warming create important new habitats, particularly suitable for studying ecosystem development where nitrogen is a limiting factor. Nitrogen availability mainly results from microbial decomposition and transformation processes, including nitrification. AOA and AOB perform the first and rate-limiting step of nitrification. Investigating the abundance and diversity of AOA and AOB is essential for understanding early ecosystem development. The dynamics of AOA and AOB community structure along a soil chronosequence in Tianshan No. 1 Glacier foreland were analyzed using qPCR and clone library methods. The results consistently showed low quantities of both AOA and AOB throughout the chronosequence. Initially, the copy numbers of AOB were higher than those of AOA, but they decreased in later stages. The AOB community was dominated by “Nitrosospira cluster ME”, while the AOA community was dominated by “the soil and sediment 1”. Both communities were potentially connected to supra- and subglacial microbial communities during early stages. Correlation analysis revealed a significant positive correlation between the ratios of AOA and AOB with soil ammonium and total nitrogen levels. These results suggest that variations in abundance and diversity of AOA and AOB along the chronosequences were influenced by ammonium availability during glacier retreat.

## 1. Introduction

Global warming has induced worldwide glacier melting and retreating [1]. Newly exposed lands represent soil chronosequences with different development stages, which are also poorly affected by human disturbance because they are usually at high altitudes and far away from cities. Hence, glacier foreland is a very suitable system for assessing microbial successions. Microorganisms play an important role in soil development, biogeochemical cycles and heterotrophic processes, improve soil nutrient availability, and facilitate vascular plant species establishment [2]. Most glacier foreland microbial studies concern the Arctic [3,4,5], Antarctica [6,7,8], Alps [9,10,11], High Andes [11,12], and the highest Asiatic mountain ranges [13,14,15,16]. These studies mainly focused on the quantities of microorganisms, microbial community structure and their variation along chronosequences, as well as relationships between plant community dynamics and soil physical factors. There are also some studies investigating an active nitrogen cycle on supra- and subglacial ecosystems [17,18,19,20,21]. However, there was a lack of functional microbial studies to address the nitrogen cycle in glacier forelands [22,23].

Glacier forelands are very barren environments with very low levels of soil carbon and nitrogen contents [2]. Nitrogen is the major limiting nutrient for ecosystem development in this pioneer habitat. There are some studies focused on the N cycle processes in glacier forelands, including N-fixation, nitrification, and denitrification. Brankatschk et al. [24] investigated the abundances and potential activities of the N-fixation process in the Damma glacier foreland and found that N-fixation activity was hardly detected and *nifH* gene copy numbers were lowest in the early stages. Duc et al. [25] confirmed this for the Damma glacier and Nemergut et al. [26] reported similar results from the Puca glacier. With this, a low level of nitrogen fixation and even lower level of denitrification and nitrification in the early stages indicated that N is a major limitation in the early stages of a glacier foreland [24]. Compared to the study of N-fixation and denitrification in glacier forelands, nitrification is relatively rarely reported in the existing studies. Nitrification is limited by ammonia conversion to nitrite by ammonia oxidizers, namely ammonia-oxidizing archaea (AOA) and bacteria (AOB). AOA and AOB highly contribute to nitrogen biogeochemical cycles, particularly in glacier ecosystems [18].

AOA and AOB community structure has been widely documented in different environments, including marine systems, soils, wastewater bioreactors, and terrestrial hot springs. Temperature, pH, salinity, fertilization regime, and ammonia availability are considered as the primary factors influencing AOA and AOB community structure and composition. Brankatschk et al. [24] reported that AOA and AOB copy numbers and potential nitrification activity in the Damma glacier foreland. However, little is known about the role of AOA and AOB in glacier foreland functioning and community composition variation along glacier retreating chronosequences.

Tianshan No. 1 Glacier is located in the Eastern Tianshan Mountains of central Asia, a dry mountain range surrounded by deserts [27]. The climate in this area is highly continental, with strong winds at high elevation [28]. Tianshan No. 1 Glacier is one of the most important glaciers in arid and semi-arid areas of central Asia, and is also considered as one among ten important glaciers in the world. Hence, it has been regularly evaluated by the World Glacier Monitoring Service (WGMS) since 1959 [29]. This is the longest and most detailed observation record in China, which makes it a suitable system for assessing microbial distribution and dynamics in relation to varying environmental conditions [30].

In a preliminary study, we found a high number of nitrifying bacteria in the glacier foreland, but did not assess their functional composition. In the present study, qPCR and clone library methods were used to analyze the variation of abundance and community structure of AOA and AOB along a soil chronosequence to address two main questions: (1) are there varying ammonia oxidizer compositions along the chronosequence and (2) are the AOA and AOB closely related to soil nutrient status at different stages of the soil chronosequence? Our study has important implications for N cycling processes and their variation in the glacier foreland.

## 2. Materials and Methods

### 2.1. Study Site and Soil Sampling

The sample sites were located at Tianshan No. 1 Glacier (N 43°06′, E 86°48′), 120 km southwest of Urumchi, China. This glacier is a northwest-facing valley glacier with two branches, the east and west branches. The glacial foreland was formed from an ongoing deglaciation event since the Little Ice Age. The annual average temperature is −5.5 °C, with positive temperature months from June to September, during which the temperature is below 5 °C, and the remaining months are negative temperature months in these areas [31]. The annual mean precipitation is 452.2 mm, with a standard deviation of 71.3 mm from 1959 to 2007, and most of the precipitation in the site occurs from June to August, accounting for 85% of the annual totals, with only small amounts falling in the winter. The lithology of moraine in the glacier foreland is mainly siliceous crystalline schist, with liberal amounts of granodiorite, gabbro, gneiss, quartzite, and granite, and occasionally, a small amount of limestone [32]. Samples were taken from the glacier foreland along the retreating trail of the east branch of Tianshan No. 1 Glacier. Soil samples with different exposure times, six different periods in total, were collected in August 2012. (Table 1). The comprehensive sampling photograph was described by Wu et al. [30]. Exposure time was based on the annual glacier retreat observation data provided by Tianshan Glaciological station of Chinese Academy of Sciences (from 1959 to 2012), and was also confirmed with lichenometric chronology data (from 1958 to 1538) [33]. Outside the moraine, the soils were ice free for more than 3000 years or annum (a), and densely covered with plants [34]. Each period’s soil samples content three quadrats, with five soil samples randomly taken in an area approximately 2 m × 2 m from each quadrat which were pooled after removing the largest gravels. Thus, there were three replicates per period. Pioneer plants were described by Wu et al. [30]. The soil samples were placed in a sterile soil box, kept frozen in ice during transport to the laboratory, stored at −20 °C and sieved (2 mm) before analyses.

### 2.2. Biogeochemical Properties of the Soil

Soil pH, organic C, and total N were analyzed following the method of Liu et al. [35]. Ammonium and nitrate were extracted with 2 M KCl solution (1:4 *w*/*v*), shaking for 1 h on a reciprocal shaker, and then the soil suspension was filtered (0.45 µm) and analyzed using flow-injection Lachat automated colorimetry system (FIAstar 5000 Analyzer, Foss, Höganäs, Sweden).

### 2.3. DNA Extraction and PCR Amplification of the amoA Gene Fragment

Soil genomic DNA was isolated using the PowerSoil DNA Isolation Kit (MoBio, Carlsbad, CA, USA) according to the manufacturer’s instructions. The concentration of DNA was determined with a NanoDrop Spectrophotometer ND-2000 (Thermo Fisher Scientific, Wilmington, NC, USA). Extracted DNA was stored at −20 °C.

The primers Arch-amoAF: (5′-STAATGGTCTGGCTTAGACG-3′) and Arch-amoAR: (5′-GCGGCCATCCATCTGTATGT-3′) [36] were used to amplify the archaeal *amoA* gene, and the primers amoA-1F: (5′-GGGGTTTCTACTGGTGGT-3′) and amoA-2R: (5′-CCCCTC(G/T)G(C/G)AAA GCCTTCTTC-3′) [37] were used to amplify the bacterial *amoA* gene. Approximately 20 ng of template DNA was used in a total volume of 50 µL PCR, containing 2 units of Taq DNA polymerase (Fermentas, Shanghai, China), 1× Taq Buffer, 3 mM MgCl_2_, 0.2 mM dNTP, and 0.4 µM each primer. The same conditions were used for bacterial and archaeal *amoA* amplification. Initial step at 94 °C for 3 min, followed by 30 cycles of 30 s denaturing at 94 °C, 30 s annealing at 55 °C, and 45 s extension at 72 °C, with a final extension step of 10 min at 72 °C. In order to avoid potential amplification biases, three replicates were performed per sample. The presence and sizes of the PCR amplification products were determined with agarose (1.0%) gel electrophoresis.

### 2.4. Cloning and Sequence Analysis of amoA Gene

The PCR products were purified using the AxyPrep DNA Gel Extraction Kit (Axygen, Union City, CA, USA) as directed by the manufacturer. Then the purified PCR products were cloned using pMD18-T Vector (Takara, Dalian, China). The ligation products were transformed into *E. coli* DH5α competent cells (Tiangen, Beijing, China) using heat-shock methods following the manufacturer’s instructions. White colonies were selected for insert screening using PCR with primers M13F (-47) and M13R (-48). For each period’s soil sample, averages of fifty positive AOA and AOB colonies were randomly selected and sequenced using the M13F primer.

Obtained DNA sequences were imported into MEGA software (Version 5.04) package to manually check sequencing errors and perform the multiple alignment [38]. In this study, operational taxonomic units (OTUs) were defined as a group of sequences differing by less than 3% using mothur software v.1.31.2 [39], with the furthest neighbor algorithm method. The representative clones of each phylotype were selected with mothur for phylogeny reconstruction, and sequences were compared with the GenBank database using the Basic Local Alignment Search Tool (BLAST) [40] in order to get the most similar sequences in the GenBank database. The phylogenetic dendrograms were constructed using the neighbor-joining method [41] with Kimura two-parameter distance, and the tree topologies were evaluated using bootstrap analysis of 1000 data sets using the MEGA5.04 package [38]. The percentage of coverage was calculated using Good’s method [42], and the rarefaction curves were obtained using DOTUR program [43].

### 2.5. Quantitative PCR Anaylsis of amoA Gene

qPCR was performed in a Stratagene MX3005p thermocycler (Agilent Technologies, La Jolla, CA, USA) with a SYBR Premix Ex Taq Ⅱ (Takara, Dalian, China). The copy numbers of archaeal and bacterial *amoA* genes were detected with primers Arch-amoAF/Arch-amoAR and amoA-1F/amoA-2R, respectively. Plasmid DNA preparation was obtained from the previously sequenced and verified archaeal and bacterial *amoA* genes clone using AxyPrep Plasmid Miniprep Kit (Axygen, Union City, CA, USA). The concentrations of plasmid DNA were quantified with a NanoDrop Spectrophotometer ND-2000 (Thermo Fisher Scientific, Wilmington, NC, USA). The copy numbers of the archaeal and bacterial *amoA* genes were calculated directly from the concentration of the extracted plasmid DNA, and ten-fold dilutions of a known copy number of the plasmid DNA were used for the standard curves, respectively.

All sample and standard reactions were carried out in triplicate. qPCR was performed in a 10 µL reaction system consisting of 5 µL SYBR Premix Ex Taq Ⅱ (Takara, Dalian, China), 0.4 µL of forward and reverse primer for each primer (archaeal and bacterial amoA, 10 µmol), 1 µL of extracted DNA (~10–25 ng), 0.2 µL ROX, and 3 µL ddH2O. The amplification program of archaeal *amoA* genes was as follows: 95 °C for 30 s; 40 cycles of 10 s at 95 °C, 25 s at 63 °C, and 45 s at 72 °C. The amplification program of bacteria *amoA* genes was as follows: 95 °C for 30 s; 40 cycles of 10 s at 95 °C, 25 s at 57 °C, and 45 s at 72 °C. Melting curve analysis was used to confirm the specificity of qPCR amplification. 

Data analysis was performed with MxPro software (Version 4.00). The C_t_ (threshold cycle) values were used to quantify the copy numbers of archaeal and bacterial *amoA* genes according to the corresponding standard curves. The standard curves of archaeal and bacterial *amoA* genes were based on ten-fold dilutions of a known copy number of the plasmid DNA, ranging from 8.07 × 10^1^ to 8.07 × 10^6^ and from 1.28 × 10^2^ to 1.28 × 10^7^ copies, respectively. Based on the average of triplicate data, the standard curves of qPCR were Y = −3.464 × log(X) + 37.71 for archael *amoA* gene and Y = −3.278 × log(X) + 35.97 for bacterial *amoA* gene, all with R^2^ values ≥ 0.995. The qPCR efficiencies for archaeal and bacterial *amoA* genes assays were 94.4% and 101.9%, respectively.

### 2.6. Statistical Analysis

Significance levels were within confidence limits of 0.05 or less. The data presented are the means of at least three independent experiments and are expressed as the mean ± SE. Comparisons between the mean values were performed using the least significant difference (LSD test) at *p* < 0.05. Correlation analysis (the two-tailed Pearson correlation coefficients) between the abundance, number of OTUs, and the diversity index of the *amoA* gene with soil physiochemical properties were also performed using SPSS 21.

## 3. Results

### 3.1. Soil Physicochemical Properties

The six periods’ soil (total of 18 soils) at the glacier foreland ranged from 8a to 3000a with pH values varying between 8.41 and 7.17. Soil organic C and total N content were low before the age of 300a, ranging from 0.286% to 1.387% and 0.026% to 0.149%. On the meadow outside the moraine, the soil organic C and total N content increased to 2.577% and 0.361%, respectively. Soil organic C and soil total N significantly increased (*p* = 0.02, *p* < 0.01) along the chronosequence and also positively correlated with the age respective (*r* = 0.897, *p* = 0.02, *r* = 0.948, *p* < 0.01). The soil ammonium content, ranging from 9.327 to 14.915 mg/kg, did not significantly (*p* = 0.25) increase along the chronosequence and showed weak positive correlation with the age (*r* = 0.561, *p* = 0.25), whereas the soil nitrate significantly increased (*p* < 0.01) and positively correlated with the age (*r* = 0.996, *p* < 0.01), ranging from 0.829 mg/kg to 4.271 mg/kg (Table 2). Soil organic C was also significant positively correlated with soil total N (*r* = 0.989, *p* < 0.01), soil ammonium (*r* = 0.835, *p* = 0.04), and soil nitrate (*r* = 0.861, *p* = 0.03).

### 3.2. Abundance of AOA and AOB

**Figure 1:**
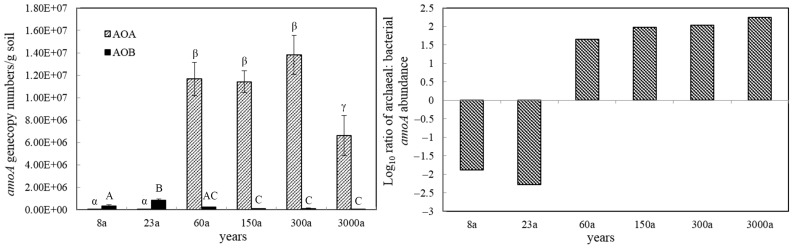
The copy numbers of *amoA* and log ratio of AOA to AOB in the Tianshan No. 1 Glacier foreland (different Greek alphabet letters indicate values that are significantly different from one another (ANOVA, *p* < 0.05) in AOA groups, and different uppercase English letters indicate values that are significantly different from one another (ANOVA, *p* < 0.05) in AOB groups).

The copy numbers of bacterial *amoA* genes in the Tianshan No. 1 Glacier foreland were very low along the chronosequence, ranging from 3.84 × 10^4^ to 8.32 × 10^5^ copies/g soil dw. The archaeal *amoA* genes copy numbers were very low in the early ages (8a–23a), ranging from 4.41 × 10^3^ to 4.75 × 10^3^ copies/g soil dw, and rapidly increased in subsequent years. The copy numbers of archaeal *amoA* genes were significantly higher than their bacterial *amoA* genes in the later ages (60a–3000a), ranging from 6.62 × 10^6^ to 1.38 × 10^7^ copies/g soil dw. The ratio of archaeal and bacterial *amoA* genes abundance in the Tianshan No. 1 Glacier foreland showed an increasing trend along the chronosequence. For example, the ratio of AOA to AOB varied from −2.28 to 2.24 along the chronosequence (Figure 1).

### 3.3. Diversity of AOA and AOB

AOA *amoA* genes copy numbers were very low (4.75 × 10^3^ and 4.41 × 10^3^ copies/g soil) at 8a and 23a, and AOB *amoA* genes copy numbers were also low in the age of 3000a (3.84 × 10^4^ copies/g soil), resulting in the fact that these clone libraries could not be successfully constructed. A total of 200 AOA clones and 250 AOB clones were randomly selected from four AOA and five AOB clone libraries and sequenced. Finally, 191 available AOA sequences and 196 AOB sequences were generated. All the *amoA* genes found in the Tianshan No. 1 Glacier foreland could be divided into 12 AOA OTUs and 27 AOB OTUs with defined group sequences differing by less than 3%. The rarefaction curves for AOA and AOB clone libraries nearly approached a plateau, suggesting that the communities were well sampled (Figure S1).

**Figure 2:**
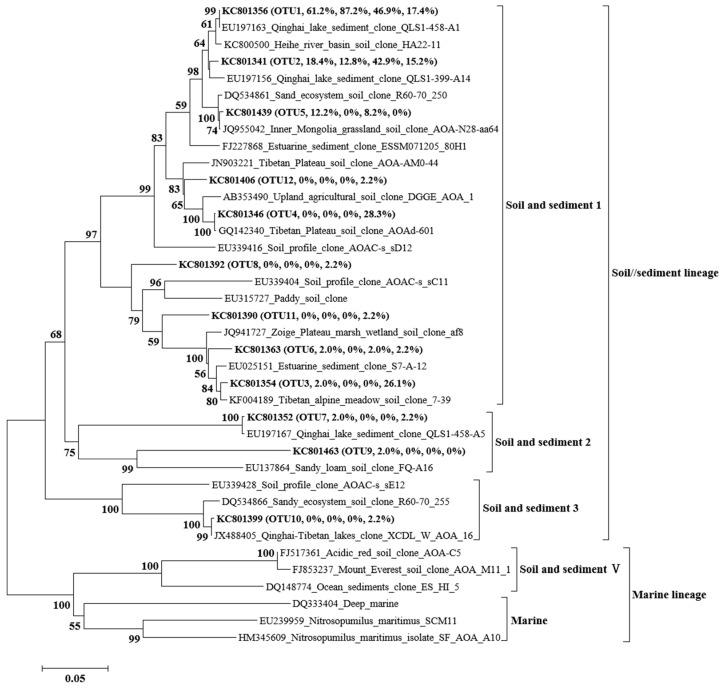
Phylogenetic analysis of archaeal *amoA* gene. Sequences obtained in this study are indicated with bold font with the accession number, followed by OTU number, then by the relative abundances of this OTU in 60a, 150a, 300a, and 3000a, respectively. The phylogenetic dendrograms were constructed using the neighbor-joining method, and the tree topologies were evaluated with bootstrap analysis of 1000 data sets using the MEGA5.04 package. Bootstrap values (>50) are indicated at branch points.

**Figure 3:**
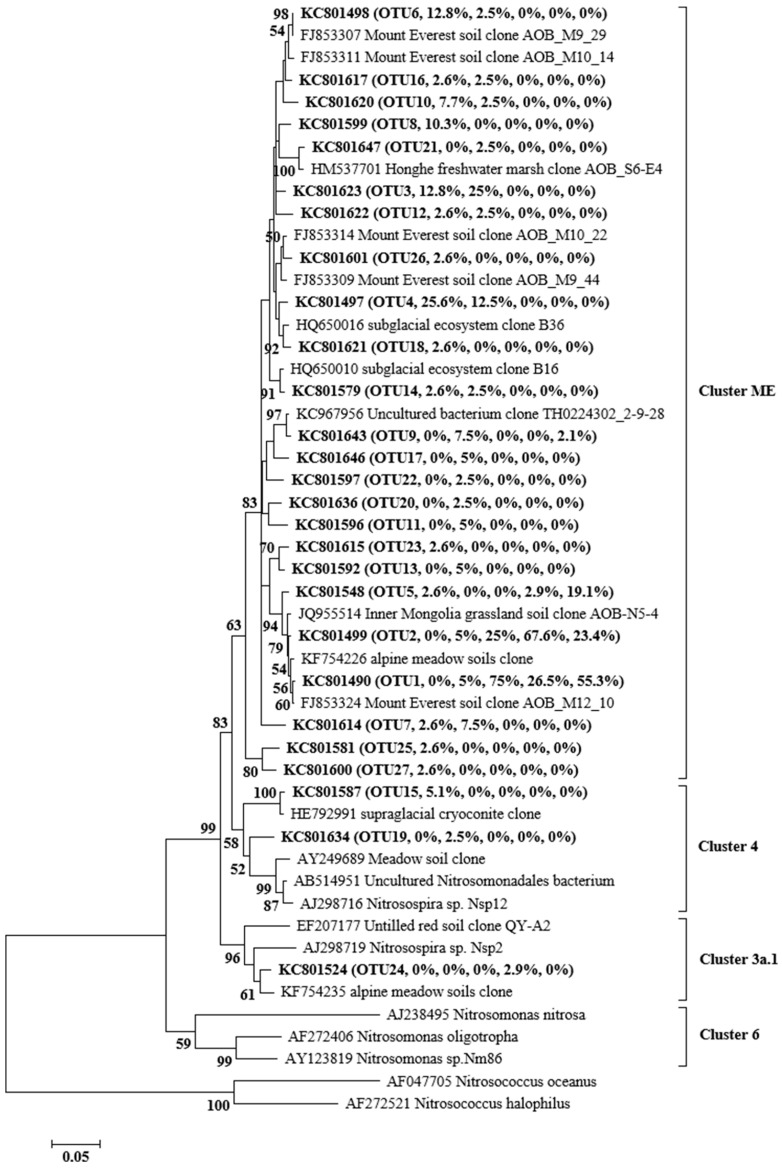
Phylogenetic analysis of bacterial *amoA* gene. Sequences obtained in this study are indicated with bold font with the accession number, followed by OTU number, then by the relative abundances of this OTU in 60a, 150a, 300a, and 3000a, respectively. The phylogenetic dendrograms were constructed using the neighbor-joining method, and the tree topologies were evaluated by performing bootstrap analysis of 1000 data sets using the MEGA5.04 package. Bootstrap values (>50) are indicated at branch points.

AOA OTU1 and AOA OTU2 were the main AOA species before the 300a, and in the 3000a, the main AOA species contained four OTUs (OTU1–4). AOB OTU3 and AOB OTU4 were the main AOB species in the early ages (8a, 23a), and AOB OTU1 and OTU2 in the later ages (60a–300a). The AOA *amoA* genes showed higher OTUs in the later age (3000a), but the AOB *amoA* genes showed higher OTUs in the early age (8a, 23a) (Figure 2 and Figure 3). The Shannon index and Simpson index showed the same trends along the chronosequence as the AOA *amoA* genes diversity index, with an upward trend, and the AOB *amoA* genes showed a downward trend (Table 3).

### 3.4. Phylogenetic Analysis of AOA and AOB

**Figure 4:**
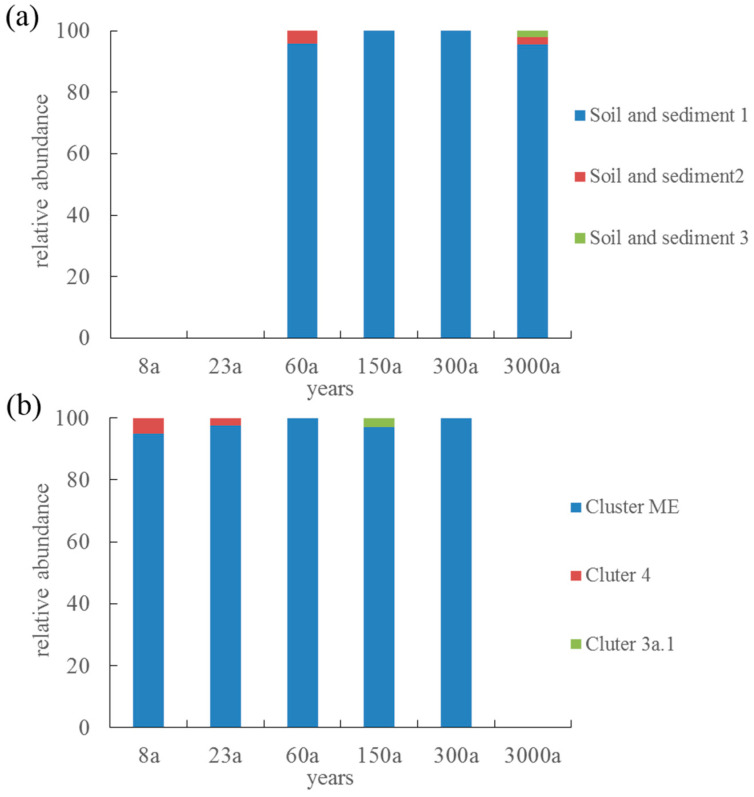
The relative abundance of different OTUs cluster of AOA (**a**) and AOB (**b**) variation along the chronosequence.

The archaeal *amoA* gene phylogenetic analysis showed that the 12 OTUs recovered from the glacier foreland could be clustered into soil/sediment lineage, including soil and sediment 1–3. The soil and sediment 1 group were the main groups in the glacier foreland, ranging from 95.7% to 100% (Figure 2 and Figure 4a). Phylogenetic analysis showed that bacterial *amoA* gene in the glacier foreland belongs to the *Nitrosospira*. The *amoA* genes could be further clustered into cluster ME, cluster 3a.1, and cluster 4 groups, and the cluster ME group was the main group in the glacier foreland, with a proportion between 97.1% and 100%, whereas the cluster 4 group only appeared in 8a and 23a, with a share ratio between 2.5% and 5.1%, and the cluster 3a.1 group only appeared in 150a, with a share ratio of 2.9% (Figure 3 and Figure 4b).

### 3.5. Relationships between Soil Physicochemical Properties and AOA and AOB

Pearson’s correlation coefficients were used to investigate the relationships between soil physiochemical properties and the abundance/diversity index of the AOA and AOB. The Simpson index of AOB was positively correlated with TOC and TN (*p* < 0.05), whereas there was no significant correlation found between the diversity index of AOA and soil physicochemical properties. The abundance of AOA and AOB also showed no significant correlation with the soil physicochemical properties, but the ratio of AOA and AOB copy numbers exhibited a significantly positive correlation with TOC, TN (*p* < 0.01), and ammonium concentration (*p* < 0.05) (Table 4).

## 4. Discussion

Low soil organic C and total N was detected in the Tianshan No. 1 Glacier foreland and significantly increased along the chronosequence. This observation could be confirmed in most glacier forelands [2]. The initial increasing could be due to a proliferation of autotrophic microorganisms, such as cyanobacteria and eukaryotic microalgae in the initial soils [44]. When the pioneer plant appeared and then the plant abundance and diversity increased along the chronosequence, plant root deposits and litter could be the major contribution [45,46]. Another contributor could be the atmospheric dry and wet deposition [24]. Outside the moraine, soil organic C and total N reached maximum values when the plants were densely covered. The soil nitrate significantly increased along the chronosequence, and soil ammonium also increased, but not significantly. This bioavailable nitrogen increase may be due to the N fixation actively increasing with the soil ages and external input, including glacier snowpack melt, aerial deposition, and the decomposition of complex organic compounds [24].

The bacterial *amoA* gene copies in the Tianshan No. 1 Glacier were very low, with around 10^4^ to 10^5^ copies/g soil. The bacterial *amoA* gene copies were higher than archaea *amoA* gene copies before the 23a, but after the 60a, the archaea *amoA* gene copies were the highest. Similar results were also found in the Damma glacier foreland [24]. This may indicate that the AOB group was the most important contribution to ammonia oxidation in the early periods (8a, 23a), and the AOA group was the most important contribution in the later ages. A possible explanation for this phenomenon could be that before the 23a, the ammonia oxidation groups may have been closely related with the subglacial sediments and cryoconites. The study about nitrifying microbial populations in the subglacial ecosystem showed that AOB were more abundant and more diverse than AOA in subglacial sediments [47]. These results are consistent with our results which show that in the early periods (8a, 23a), AOB were predominant. Segawa et al. [17] studied the nitrogen cycle in cryoconites at the same site as us and found an amount of bacterial *amoA* gene but hardly detected archaeal *amoA* gene. In the early periods, soil microbial community and soil nutrient contents may have possibly been transported from cryoconite either through the sub-glacier environment or directly from the glacier surface during ice melting. Hamilton et al. [47] reported that microbial community structure from cryoconites, sub-glaciers, and surface snow were connective with similar phylogenetic composition. Our results indicate that the AOA and AOB community potentially linked with supra- and subglacial microbial communities in the early periods of the glacier foreland.

Interestingly, we observed the occurrence of a switch in the ratio of the AOA and AOB along the succession age. Pearson correlation analysis showed an AOA abundance and the diversity index showed no significant correlation with the soil’s physicochemical properties, while the AOB Simpson index showed a significant positive correlation with TOC and TN. Because TOC and TN were positively correlated (*r* = 0.989, *p* < 0.01), and both AOA and AOB are thought to be autotrophic [48], this suggests that the AOB community changed from homogeneous to heterogeneous with an increase in TN. Several studies showed that N might have closed relationship with the AOB community composition, as the N gradient significantly changed AOB community compositions [49,50]. Pearson correlation analysis also exhibited the ratio of AOA and AOB copy numbers as significantly positive with TOC, TN, and ammonium concentration. In fact, the ratio of AOA and AOB directly correlate with TN and ammonium concentration. AOB usually exhibited high activity with a high availability of ammonia, but for the growth of AOA, low availability of ammonia in the environment [51,52,53] is preferable. The different affinity to ammonia of AOB and AOA might lead the copy numbers of AOA to be greater than those of AOB after 60a. This result shows that in the late succession age, the AOA group played a vital contribution to ammonia oxidation in the foreland, consistent with other studies showing that the AOA group is the dominant group in continent soils [48,54,55,56]. This result indicated that the availability of ammonium along the glacier retreat sequence caused AOA and AOB variation along the chronosequence.

Environmental AOB mainly belonged to β- and γ-Proteobacteria [57]. The γ-Proteobacterial AOB is suitable for growth in a marine environment [58,59], while β-Proteobacterial AOB is usually found in soil and freshwater environments. The β-Proteobacteria AOB were divided into *Nitosomonas* and *Nitrosospira* genera [60], which normally favor high-ammonia and low-ammonia environments, respectively [61]. All AOB sequences obtained from the Tianshan No. 1 Glacier foreland fell within *Nitrosospira* cluster 4, cluster 3a.1, and cluster ME, which was first reported by Zhang et al [62]. These results are consistent with the dominance of *Nitrosospira* within the AOB group in soils [63,64]. Other works conducted in agricultural and forest soils showed that *Nitrosospira* cluster 2 and cluster 4 were the main groups, while *Nitrosospira* cluster 1 and cluster 3a were mainly distributed in the ammonia-rich soils [54,65]. In low-ammonia, unimproved soils, cluster 4 is the main group, while cluster 2 group has a relatively high abundance in acidic soils [66]. This is consistent with our study, in which cluster 4 appeared in low-ammonia stages (8a–23a). When the ammonia significantly increased after 150a, the cluster 3a.1 group appeared. In our study, *Nitrosospira* cluster ME was the dominant group in the glacier foreland, consistent with Zhang et al. [62] who showed that cluster ME was the dominant AOB group at a high altitude on Mount Everest. This suggests that members of cluster ME are suited to survive at high altitudes in cold and oligotrophic environments. 

All AOA sequences fell within soil/sediment lineages dominated by the soil and sediment 1, suggesting that AOA communities in these soils are distinct from those in aquatic environments. Several studies showed that the external input of nitrogen into alkaline soil did not drive AOA community changes [55,65,67], but that it changed the AOA community structure in acidic soils [49]. There was a nutrient gradient along the glacier retreat sequence, but the cluster of the AOA community was relatively stable, likely due to the high pH of the glacier foreland soils. 

## 5. Conclusions

Our data revealed that the number of ammonia oxidizers in glacier forelands is relatively lower than in other environments. AOB made a vital contribution to ammonia oxidation in the early ages, but AOA became more important in the later ages. The AOB community was dominated by cluster ME, which indicates that cluster ME is suited to survive at high altitudes in cold and oligotrophic environments. AOB diversity decreased along the chronosequence, but AOA diversity remained relative stable; this may be due to the different affinity to ammonia of AOB and AOA. In the early periods, AOA and AOB communities potentially linked with supra- and subglacial microbial communities, and the abundance of AOA and AOB shifted along the chronosequence, suggesting that ammonium availability influences AOA and AOB abundance. But the mechanism needs further study in the future.

## Figures and Tables

**Table 1:** Distance from the glacier front and retreated time of the sample.

Distance from the Glacier Front	15 m	34 m	250 m	450 m	600 m	750 m
Retreated time	8a	23a	60a	150a	300a	3000a

**Table 2:** Soil physicochemical properties along the chronosequence.

Distance from the Glacier Front (m)	Soil Age after Deglaciation (years)	pH	Total Organic C (% dw)	Total N (% dw)	Ammonium (mg/kg dw)	Nitrate (mg/kg dw)
15	8a	8.41 ± 0.25 ^a^	0.286 ± 0.009 ^a^	0.026 ± 0.006 ^a^	10.516 ± 0.036 ^a^	1.031 ± 0.017 ^a^
34	23a	7.75 ± 0.17 ^b^	0.454 ± 0.067 ^a^	0.055 ± 0.008 ^a^	9.327 ± 0.143 ^a^	0.961 ± 0.095 ^a^
250	60a	7.70 ± 0.03 ^b^	0.524 ± 0.065 ^a^	0.040 ± 0.007 ^a^	9.512 ± 0.038 ^a^	0.829 ± 0.149 ^a^
450	150a	7.17 ± 0.21 ^b^	1.229 ± 0.019 ^b^	0.129 ± 0.003 ^b^	12.996 ± 0.056 ^b^	0.925 ± 0.067 ^a^
600	300a	7.32 ± 0.26 ^b^	1.387 ± 0.038 ^b^	0.149 ± 0.007 ^b^	14.915 ± 0.106 ^b^	1.051 ± 0.094 ^a^
750	3000a	7.37 ± 0.26 ^b^	2.577 ± 0.281 ^c^	0.361 ± 0.045 ^c^	14.427 ± 0.406 ^b^	4.271 ± 1.082 ^b^

Results are given as means ± SE (*n* = 3). Different superscript letters indicate values that are significantly different from one another (ANOVA, *p* < 0.05).

**Table 3:** The coverage and diversity index of *amoA* genes libraries.

Succession Years	OTUs		Coverage		Shannon Index		Simpson Index	
	AOA	AOB	AOA	AOB	AOA	AOB	AOA	AOB
8a	ND	16	ND	74.4%	ND	2.40	ND	0.88
23a	ND	18	ND	77.5%	ND	2.57	ND	0.89
60a	7	2	91.8%	100.0%	1.19	0.56	0.57	0.38
150a	2	4	100.0%	94.1%	0.19	0.82	0.22	0.47
300a	4	4	98.0%	97.9%	1.00	1.07	0.59	0.60
3000a	10	ND	87.0%	ND	1.80	ND	0.80	ND

ND: not detected.

**Table 4:** Pearson correlation between soil physiochemical properties, *amoA* copies, and diversity index.

	OTU		Shannon Index		Simpson Index		Copy Numbers		Copy Numbers Ratio
	AOA	AOB	AOA	AOB	AOA	AOB	AOA	AOB	AOA/AOB
Year	0.770	−0.518	0.754	−0.625	0.679	−0.804	0.020	−0.478	0.792
TOC	0.735	−0.702	0.745	−0.755	0.761	−0.833 *	0.364	−0.672	0.968 **
TN	0.728	−0.621	0.732	−0.695	0.719	−0.814 *	0.229	−0.591	0.923 **
pH	−0.455	0.710	−0.449	0.690	−0.574	0.604	−0.731	0.491	−0.762
Ammonium	0.434	−0.619	0.510	−0.586	0.614	−0.539	0.540	−0.762	0.876 *
Nitrate	0.727	−0.446	0.710	−0.562	0.621	−0.758	−0.067	−0.426	0.739
C/N ratio	−0.100	−0.020	−0.138	0.054	−0.120	0.217	0.226	−0.015	−0.501
A/N ratio	−0.534	0.078	−0.493	0.227	−0.326	0.493	0.462	0.018	−0.332

TOC, total organic carbon; TN, total nitrogen; C/N ratio, the ratios of total organic carbon to total nitrogen; A/N ratio, the ratios of ammonium to nitrate. ** *p* < 0.01; * *p* < 0.05.

## Data Availability

All *amoA* gene sequences obtained from the Tianshan No. 1 Glacier foreland were clustered into unique sequences (100% DNA sequence similarity level) with the average neighbor algorithm implemented in the software mother v.1.31.2 (Schloss et al. 2009). Representative sequences of novel genes sequences from the glacier foreland of this study were deposited in GenBank database under accession numbers KC801341-KC801488 for archaeal *amoA* gene and KC801489-KC801510, KC801512-KC801522, KC801524-KC801559, KC801561-KC801650 for bacterial *amoA* gene.

## Supplementary Materials

The following supporting information can be downloaded at: https://www.mdpi.com/article/10.3390/microorganisms11122871/s1, Figure S1: Rarefaction curves of OTUs in AOA clone libraries (a) and AOB clone libraries (b). The reference line represents 1:1, indicating infinite diversity.
