# Supplementary figures and images for: Changes in Diversity and Abundance of Ammonia-Oxidizing Archaea and Bacteria along a Glacier Retreating Chronosequence in the Tianshan Mountains, China
Source: Microorganisms. 2023 Nov 27;11(12):2871. doi: 10.3390/microorganisms11122871 (PMC10745509; doi:10.3390/microorganisms11122871)

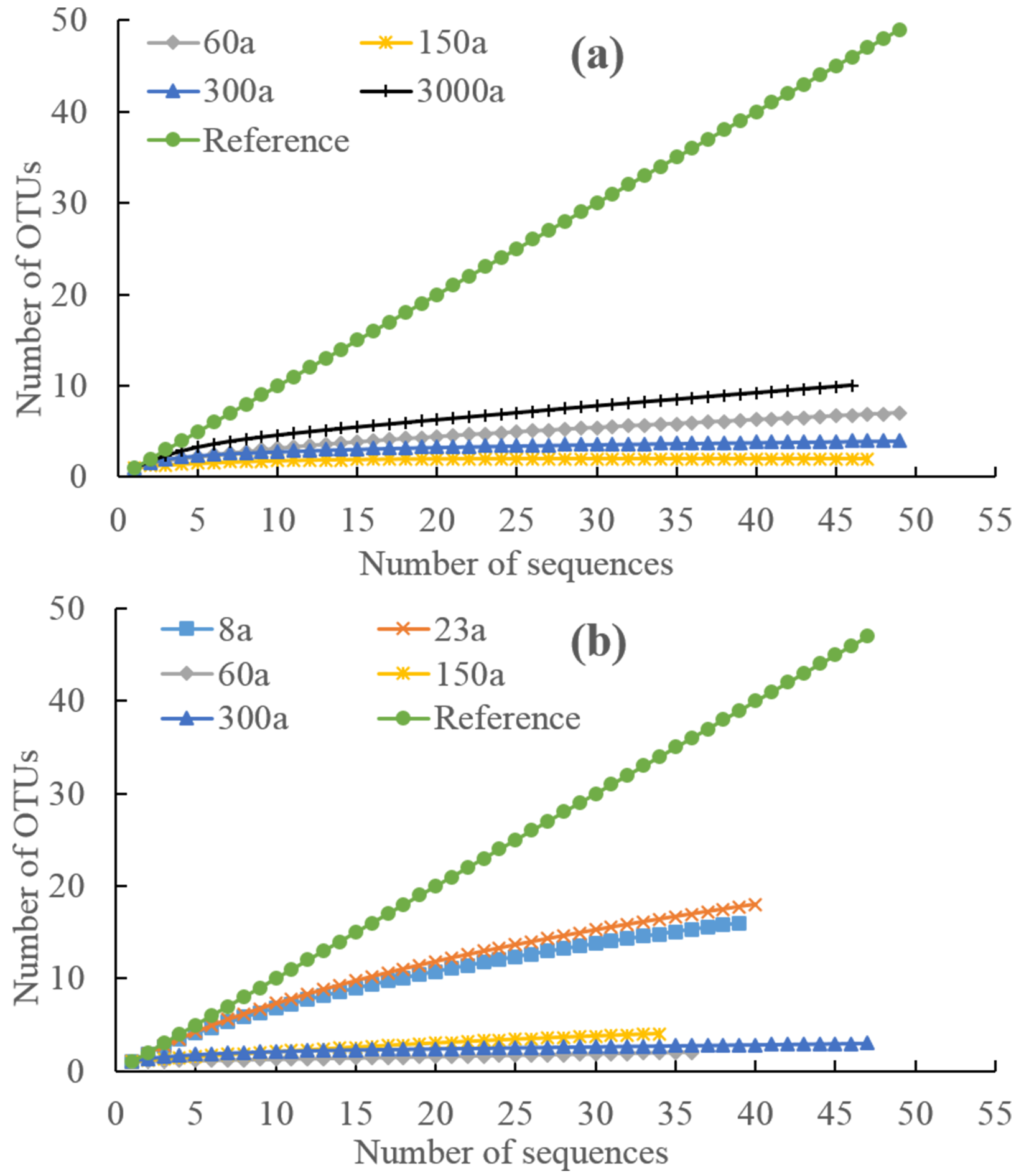

Supplement: Supplementary file 1 [file microorganisms-11-02871-s001.zip › Figure S1.tif]
